# Associations between spanking beliefs and reported spanking among adolescents-parent/caregiver dyads in a Canadian sample

**DOI:** 10.1186/s12889-022-12856-z

**Published:** 2022-03-12

**Authors:** Tracie O. Afifi, Samantha Salmon, Ashley Stewart-Tufescu, Tamara Taillieu, Janique Fortier, Harriet MacMillan, Joan Durrant, George W. Holden

**Affiliations:** 1 Departments of Community Health Sciences and Psychiatry and Scientist, Children’s Hospital Research Institute of Manitoba, University of Manitoba, S113-750 Bannatyne Avenue, Winnipeg, Manitoba R3E 0W5 Canada; 2 Department of Community Health Sciences, University of Manitoba, Winnipeg, Canada; 3 Faculty of Social Work, and Scientist, Children’s Hospital Research Institute of Manitoba, University of Manitoba, Winnipeg, Canada; 4 Departments of Psychiatry and Behavioural Neurosciences, and of Pediatrics, McMaster University, Hamilton, Canada; 5 Department of Psychology, Southern Methodist University, Dallas, TX USA

**Keywords:** Physical punishment, Corporal punishment, Child maltreatment, Spanking, Spanking beliefs, Hitting, Prevention

## Abstract

**Background:**

Research consistently demonstrates that physical punishment of children including “spanking” is harmful. Interest in effective prevention is growing rapidly. The aim of the current study is to examine spanking beliefs among adolescents and parents in relation to reports of spanking that the adolescents experienced before 11 years of age.

**Methods:**

Data were drawn from Wave 1 of a study conducted in 2017–2018 that included adolescents (14–17 years old) and one of their parents/caregivers from Manitoba, Canada (*n* = 1000 pairs). The study objectives were to examine: 1) spanking beliefs of adolescents and their parents; 2) the correlation between parent and adolescent spanking beliefs; 3) whether parents perceive the words “spank” vs. “hit” differently using intraclass correlation; 4) the association between parents’ beliefs about spanking and parent- and adolescent-reported use of it; and 5) the relationship between sociodemographic variables and spanking. The data were analyzed using descriptive statistics, Spearman’s correlation, intraclass correlation, and binary and multinomial logistic regression analyses.

**Results:**

The prevalence of adolescent-reported and parents’-reported spanking were 46.0% and 39.6%, respectively. The proportions agreeing that spanking is a normal part of parenting were similar among adolescents (22.0%) and parents (18.5%), and were moderately correlated (intraclass correlation = 0.38, SE = 0.038). More than five times as many parents believed that “spanking” is necessary (19.5%) than believed that “hitting” is necessary (3.5%). Parents’ positive spanking beliefs were associated with increased likelihood of adolescent- and parent-reported spanking. Few significant associations were found between sociodemographic variables and parent-reported or adolescent-reported spanking.

**Conclusions:**

Adolescents’ spanking beliefs are related to their parents’ spanking beliefs, suggesting that they are transmitted across generations. Public education and law reform are needed to decrease the normalization and perceived necessity of spanking in child-rearing. Efforts should include improving the understanding that spanking is a form of violence against children. With only a few significant differences noted between sociodemographic variables and parent- and adolescent- reported spanking and the prevalent use of spanking across all sociodemographic variable categories, it may be useful to develop universal approaches to awareness-raising and implementation of education strategies in Canada.

## Background

Globally, the most pervasive forms of violence against children are physical and emotional punishment committed by their parents and other caregivers [1–5]. Over the past two decades, mounting evidence indicates that physical (also called corporal) punishment is harmful to children and has no known benefits [6–14]. It has consistently been shown to be a risk factor for injury, aggression, anti-social behavior, mental health problems, poor parent–child relationships, slower cognitive development, and violence towards partners and children later in life [6–14]. In 2018, the American Academy of Pediatrics published a statement explicitly recommending against any physical or emotional punishment, including spanking, hitting, slapping, threatening, insulting, humiliating, and shaming [16]. Similarly, in 2019, the Canadian Paediatric Society published a position statement stating, “At no time should parents use physical punishment – spanking, slapping, hitting – or behaviour that shames children” [17].

At the global level, efforts to end physical punishment of children have been underway for half a century. The United Nations (UN) Convention on the Rights of the Child (CRC) guarantees children protection from “all forms of physical or mental violence … while in the care of parent(s), legal guardian(s) or any other person who has the care of the child” [18]. The UN Committee on the Rights of the Child has explicitly identified physical punishment as a form of violence, calling its elimination “a key strategy for reducing and preventing all forms of violence in societies” [19]. The Committee has called on all State parties to the CRC to monitor their progress towards eliminating physical punishment, and to conduct research with children and their parents/carers to assess its prevalence and attitudes toward it [19]. Most recently, the UN 2030 Agenda for Sustainable Development set a target of eliminating all forms of violence against children (Target 16.2) [20]. In recognition of children’s rights to be protected from violence including corporal punishment, and in support of the 2030 Sustainable Development Agenda, there has been an increased need for prevalence data and evidence that can inform effective prevention strategies [20].

Prevalence estimates indicate that physical punishment remains a common experience of childhood around the world. For example, data from 49 low- and middle-income countries indicate that approximately 62.5% of young children (aged 2 to 4 years old) have experienced punishment [21]. To assess shifts in prevalence over time, the best data would come from population-level longitudinal or cohort data using the same methods and measures over time. Unfortunately, such data are rare. However, evidence from cross-sectional surveys from Canada and the United States suggests that physical punishment is decreasing over time [2, 22–26]. Recent cross-sectional data from the United States indicates that 49% of children (below 10 years of age) and 23% of adolescents (10 to 17 years old) were spanked in 2014 [25]. In Québec, using survey data, 34.7% of adult/caregiver respondents reported using physical punishment in 2012 [2].

In the face of continually mounting evidence of its risks, physical punishment of children has been fully prohibited in 63 countries [27]. However, Canada and the United States are not yet among those countries. Across North America, physical punishment remains a lawful parental response to conflict with children despite mounting evidence of its risks and declining support for its use. Because a large number of caregivers worldwide still report using physical punishment and it has been consistently identified as a risk factor in children’s physical and psychological development, its prevention should be a public health priority. To achieve this aim, further research is needed to inform effective violence prevention efforts targeting a reduction in physical punishment.

A primary predictor of parents’ use of corporal punishment is the belief that it is a normal, necessary, and expected part of parenting [28–32]. In a Canadian study, approval of physical punishment was found to be the most powerful of eight predictors of mothers’ use of physical punishment with their preschoolers [30]. A key belief among mothers at high risk for using punishment is that physical punishment is necessary and instrumental for achieving parental goals [33]. Furthermore, data from 65 countries found that the caregiver’s belief that physical punishment was needed for a child to be raised properly was associated with the largest odds of spanking (Odds Ratio = 2.55, *p* < 0.001) [34]. Throughout the literature, supportive attitudes toward physical punishment are consistently associated with an increased likelihood of its use [30–32].

In addition to legislative bans, a key approach for decreasing physical punishment may be to shift adolescents’ attitudes. It is likely that adolescents’ beliefs about physical punishment are linked to their parents’ beliefs, but few studies have examined that relationship. To date, most of the research on physical punishment has relied on parent samples who self-report use of physical punishment [9, 13]. As well, research on spanking attitudes or beliefs has mostly focused on adults—both parents and professionals [32, 35–38]. Very little is known about the spanking beliefs of adolescents. One study that assessed adolescents’ attitudes toward physical punishment was published almost 20 years ago [39]. It found that adolescents who had been spanked by their mothers were more accepting of it than other adolescents. However, the attitudes of their parents were not examined. It is likely that adolescents’ beliefs about physical punishment are linked to their parents’ beliefs, but few studies have examined that relationship. Understanding how parental beliefs about physical punishment relate to adolescent beliefs can provide important insight into intergenerational cycles of violence, and can inform prevention efforts. Adolescence might be a critical developmental period preceding parenthood to intervene in an effort to reduce support for physical punishment, thus interrupting the intergenerational cycles of punitive violence against children. Adolescent development is marked by the process of individuation, whereby the adolescent becomes increasingly independent from parents and concrete thinking progresses to increasingly abstract and complex abstract thinking [40]. It is possible that efforts to reduce supportive or normative beliefs about spanking during this formative developmental period may correspond with less use of physical punishment when these individuals become parents themselves.

It would also be informative to understand how the language used to describe physical punishment may be related to parental beliefs about its use. The word “spank,” which has been adopted in North America as a euphemism for “hit”, tends to dissociate the act from ‘violence’ or ‘abuse’ and contributes to its acceptability [41]. Other euphemisms for hitting, which also include “smacking”, “slapping” and “tapping” children, are used to normalize and minimize the experience of physical punishment [42]. Furthermore, it has been found that the specific verb used to refer to physical punishment alters the perception of it, with “spank” rated as most acceptable followed by “swat,” “hit,” “slap,” and “beat” [43]. It has even been recommended that child maltreatment professionals only use terms such as “hitting” in an effort to condemn rather than support the use of physical punishment [44]. This implies that the language we use may influence support for and use of spanking; this information could be important in determining prevention strategies and warrants further examination.

The purpose of this study was to examine in a Canadian sample: 1) beliefs about spanking among adolescents and their parents; 2) the correlation between parent and adolescent spanking beliefs; 3) whether parents perceive the words ‘spank’ and ‘hit’ differently; 4) the association between parents’ beliefs about spanking and parent- and adolescent-reported use of it; and 5) the relationship between sociodemographic variables and spanking.

## Method

### Study design and participants

The Well-Being and Experiences (WE) Study is a longitudinal, intergenerational cohort study of the health and well-being of adolescents in Winnipeg, Manitoba and surrounding communities. Winnipeg is the largest city in the province of Manitoba with a population of approximately 753,700. The present study uses data collected at Wave 1 (baseline) from (*N* = 1,000) adolescents (aged 14 to 17 years old) and parent/caregiver dyads between July 2017 and October 2018. Participants were recruited to participate through random digit dialing (21.0%), referrals (40.6%), and community advertisements (38.4%). Few differences in sample characteristics were noted based on method of recruitment [45]. Forward Sortation Area (first three digits) from postal codes, adolescent sex, household income, and ethnicity were monitored to ensure the sample was similar to the population from which it was drawn [46]. The adolescent and the parent/caregiver most knowledgeable about the selected adolescent (85% were birth, step-, or adoptive mothers; 13% were birth, step-, or adoptive fathers; 2% were other caregivers; hereafter referred to as “parents”) completed separate self-administered questionnaires at a research facility in private rooms. Parents could not review adolescent responses and vice versa. Adolescents and parents provided informed consent to participate in the study in accordance with the ethics approval that was provided from the Health Research Ethics Board at the University of Manitoba. Stata version 16.1 was used to analyze the data.

### Measures

#### Beliefs about spanking

To assess adolescents’ and their parents’ beliefs that spanking is a normal part of parenting, they were asked to indicate their level of agreement on a five-point scale (strongly agree, agree, neither agree nor disagree, disagree, strongly disagree) with the statement, “Spanking is a normal part of parenting.” This item is commonly used to examine spanking beliefs [31]. In addition, parents were asked to rate the following statements on the same five-point scale: 1) “Some children need to be spanked so that they will learn a lesson,” and 2) “Some children need to be hit so that they will learn a lesson.” Parents’ responses to the three items were recoded into three categories (strongly agree/agree, neither agree nor disagree, and disagree/strongly disagree) to ensure adequate statistical power for the analyses.

#### Spanking

Adolescents were asked whether parents or caregivers ever spanked their bottom (bum) with a hand when they were 10 years of age or younger. Parents were asked whether the adolescent participant was ever spanked by any parent or caregiver with a hand on the bottom (bum) when the child was 10 years of age or younger. Response options to both items were “yes” or “no”.

#### Demographic characteristics

Self-reported total household income received by all household members, from all sources, before taxes and deductions in the past 12 months was collected from the parent at Wave 1 and coded into the following categories: $49,999 or less, $50,000 to $99,999, $100,000 to $149,999, and $150,000 or more. The parent also reported their highest level of education: high school completion or less, some community post-secondary education without graduating, completed trade school or community college, completed a university undergraduate degree, and completed a university graduate degree. Adolescent sex was reported as male or female.

### Statistical analysis

First, descriptive statistics were used to examine spanking and spanking beliefs by sociodemographic variables. Second, the strength of adolescents’ and parents’ spanking beliefs and their reports of whether spanking had occurred were computed. Third, Spearman’s correlation was computed on parents’ responses to the items asking parents about the necessity of “spanking” and “hitting”. Fourth, the intraclass correlation between parent and adolescent spanking beliefs was computed. Fifth, multinomial logistic regression models were computed to determine the association between parents’ spanking beliefs and adolescents’ spanking beliefs while adjusting for adolescent sex, household income, and parent education. Sixth, logistic regression models were computed to determine the association between parents’ spanking beliefs and parent- and adolescent-reported spanking while adjusting for adolescent sex, household income, and parent education. Finally, logistic regression models were computed to determine whether adolescent sex, household income, and parent education (entered together in one model) were associated with a) parent- and b) adolescent-reported spanking. Assumptions of multinomial and logistic regression were assessed, and it was confirmed that no violations existed. Missing data were low and, therefore, complete case analysis was used for all models.

## Results

Similar proportions of adolescents (22.0%) and parents (18.5%) agreed that spanking is a normal part of parenting. Adolescents’ and parents’ ratings on this item were significantly correlated (Intraclass correlation = 0.38; standard error [SE] = 0.038). Almost half of the adolescents (46.0%) reported being spanked as children, while 39.6% of parents reported that their adolescent was spanked as a child. Adolescents’ and parents’ responses to this item were significantly correlated (Intraclass correlation = 0.45; SE = 0.045). Among parents, 19.5% agreed that “some children need to be spanked so they will learn a lesson;” 3.5% agreed that “some children need to be hit so they will learn a lesson”.

Table 1 provides descriptive statistics for parent-reported spanking beliefs and parent reports of spanking by sociodemographic characteristics. Table 2 provides the prevalence of adolescent-reported spanking beliefs and of ever been spanked by sociodemographic variables.

**Table 1:** Parents’ spanking beliefs and parents’ reports of whether their child was spanked by their sociodemographic characteristics

	Parent-reported: Spanking is a normal part of parenting (*n* = 975)				Parent-reported: Some children need to be *spanked* so that they will learn a lesson (*n* = 962)				Parent-reported: Some children need to be *hit* so that they will learn a lesson (*n* = 977)				Parent-reported: Child ever spanked, age 10 or younger (*n* = 959)	
	Disagree % (n)	Neither % (n)	Agree % (n)	Disagree % (n)		Neither % (n)	Agree % (n)	Disagree % (n)		Neither % (n)	Agree % (n)	No % (n)		Yes % (n)
**Total Sample**	54.9 (535)	26.7 (260)	18.5 (180)	58.1 (559)		22.4 (215)	19.5 (188)	89.3 (872)		7.3 (71)	3.5 (34)	60.4 (579)		39.6 (380)
**Adolescent Sex**														
Male	47.1 (251)	47.1 (122)	53.9 (97)	46.7 (260)		45.3 (97)	54.8 (103)	48.2 (419)		43.7 (31)	67.7 (23)	46.7 (270)		50.5 (191)
Female	52.9 (282)	52.9 (137)	46.1 (83)	53.3 (297)		54.7 (117)	45.2 (85)	51.8 (450)		56.3 (40)	32.4 (11)	53.3 (308)		49.5 (187)
**Household Income**														
$49,999 or less	20.2 (103)	22.5 (55)	22.0 (38)	20.0 (107)		20.6 (41)	23.5 (43)	20.3 (168)		21.7 (15)	36.4 (12)	20.1 (109)		21.6 (80)
$50,000 to $99,999	32.5 (166)	37.7 (92)	46.8 (81)	32.1 (172)		39.7 (79)	45.4 (83)	35.6 (295)		42.0 (29)	45.5 (15)	34.1 (185)		40.4 (150)
$100,000 to $149,999	24.5 (125)	23.4 (57)	20.8 (36)	25.2 (135)		22.6 (45)	20.2 (37)	24.4 (202)		18.8 (13)	xxx	24.1 (131)		22.9 (85)
$150,000 or more	22.9 (117)	16.4 (40)	10.4 (18)	22.8 (122)		17.1 (34)	10.9 (20)	19.8 (164)		17.4 (12)	xxx	21.7 (118)		15.1 (56)
**Parent Education**														
High school completion or less	10.3 (55)	20.4 (53)	16.1 (29)	12.0 (67)		19.5 (42)	14.9 (28)	14.1 (123)		12.7 (9)	14.7 (5)	14.0 (81)		14.0 (53)
Some post-secondary	10.3 (55)	15.4 (40)	11.1 (20)	11.1 (62)		13.0 (28)	10.1 (19)	11.5 (100)		12.7 (9)	xxx	10.2 (59)		14.0 (53)
Completed trade school or community college	21.9 (117)	28.1 (73)	25.0 (45)	22.0 (123)		28.4 (61)	27.1 (51)	24.7 (215)		21.1 (15)	29.4 (10)	23.0 (133)		26.3 (100)
Completed a university undergraduate degree	28.8 (154)	21.2 (55)	19.4 (35)	28.3 (158)		20.9 (45)	19.7 (37)	25.3 (221)		26.8 (19)	xxx	25.2 (146)		25.0 (95)
Completed a university graduate degree	28.8 (154)	15.0 (39)	28.3 (51)	26.7 (149)		18.1 (39)	28.2 (53)	24.4 (213)		26.8 (19)	38.2 (13)	27.6 (160)		20.8 (79)

xxx indicates low cell counts. These cells have been blocked

**Table 2:** Adolescents’ spanking beliefs and their reports of being spanked by their sociodemographic characteristics

	Adolescent-reported: Spanking is a normal part of parenting (*n* = 966)			Adolescent-reported: Ever spanked, age 10 or younger (*n* = 919)	
	Disagree % (n)	Neither % (n)	Agree % (n)	No % (n)	Yes % (n)
**Total Sample**	49.4 (477)	28.7 (277)	22.0 (212)	54.0 (496)	46.0 (423)
**Adolescent Sex**					
Male	42.5 (202)	49.6 (137)	56.6 (120)	48.2 (238)	46.5 (196)
Female	57.5 (273)	50.4 (139)	43.4 (92)	51.8 (256)	53.6 (226)
**Household Income**					
$49,999 or less	16.6 (75)	23.5 (63)	26.1 (52)	20.7 (97)	19.7 (80)
$50,000 to $99,999	33.0 (149)	39.2 (105)	41.2 (82)	30.9 (145)	43.1 (175)
$100,000 to $149,999	25.7 (116)	23.1 (62)	19.6 (39)	25.2 (118)	22.7 (92)
$150,000 or more	24.8 (112)	14.2 (38)	13.1 (26)	23.2 (109)	14.5 (59)
**Parent Education**					
High school completion or less	10.1 (48)	18.4 (51)	16.1 (34)	13.3 (66)	14.5 (61)
Some post-secondary	10.9 (52)	10.5 (29)	14.7 (31)	8.5 (42)	14.3 (60)
Completed trade school or community college	23.3 (111)	23.8 (66)	25.6 (54)	22.2 (110)	24.7 (104)
Completed a university undergraduate degree	29.8 (142)	22.7 (63)	17.1 (36)	30.7 (152)	20.4 (86)
Completed a university graduate degree	25.8 (123)	24.6 (68)	26.5 (56)	25.4 (126)	26.1 (110)

Table 3 provides crosstabulation data and chi-square tests of significance (X^2^ [df] = 294.0 (4), *p* < 0.0001) on parent-reported beliefs that “some children need to be *hit* so that they will learn a lesson” and that “some children need to be *spanked* so that they will learn a lesson.” Of those who reported agreeing that “some children needed to be *hit* to learn a lesson,” 100% also reported agreeing that “some children needed to be *spanked* to learn a lesson.” Of those who reported neither agreeing or disagreeing with the statement that “some children needed to be *hit* to learn a lesson,” 76.1% also reported neither agreeing nor disagreeing that “some children need to be *spanked* to learn a lesson,” while 23.9% reported agreeing with this statement. Of those parents who reported disagreeing that “some children need to be *hit* to learn a lesson,” 65.5% also disagreed that “some children need to be *spanked* to learn a lesson,” while 18.6% neither agreed nor disagreed and 15.8% agreed with this statement. The association between parents’ beliefs that some children need to be “spanked” and their beliefs that some children need to be “hit” was significant (Spearman’s correlation = 0.41, *p* < 0.01).

**Table 3:** Cross-tabulation of parents’ beliefs in the necessity of spanking versus hitting

	Parent-reported: Some children need to be hit so that they will learn a lesson			
	Disagree %	Neither %	Agree %	X^2^ (df)
Parent-reported: Some children need to be spanked so that they will learn a lesson				
Disagree	65.5	0.0	0.0	294.0 (4) ***
Neither	18.6	76.1	0.0	
Agree	15.8	23.9	100.0	

^*^ p < .05; ** *p* < .01; *** *p* < .001

Table 4 reports associations between parent- and adolescent-reported spanking beliefs. Parents’ agreement that spanking is a normal part of parenting was associated with increased odds that adolescents would either hold neutral beliefs (neither agree nor disagree) (Adjusted Risk Ratio [ARR] = 3.84, 95% Confidence Interval [CI] = 2.43 to 6.08) or “agree” that spanking is normative (ARR = 7.09, 95% CI = 4.38 to 11.49). Parents’ beliefs that “some children need to be spanked” and that “some children need to be hit” were both associated with increased odds that their adolescent children would believe that spanking is normative (ARR were 4.97 and 2.91, respectively).

**Table 4:** Associations between parents’ and adolescents’ beliefs about the normativity of spanking

Parent-reported Spanking Beliefs	Adolescent-reported: Spanking is a normal part of parenting				
	Disagree	Neither	Agree	Neither vs Disagree (ref)	Agree vs Disagree (ref)
	%	%	%	ARRR (95% CI)	ARRR (95% CI)
Spanking is a normal part of parenting					
Disagree (ref)	70.6	43.1	35.6	1.00	1.00
Neither	20.0	33.8	31.7	2.60 (1.78–3.78) ***	2.98 (1.92–4.61) ***
Agree	9.4	23.1	32.7	3.84 (2.43–6.08) ***	7.09 (4.38–11.49) ***
Some children need to be spanked so that they will learn a lesson					
Disagree (ref)	72.0	48.1	39.4	1.00	1.00
Neither	16.3	30.2	27.8	2.74 (1.85–4.05) ***	2.87 (1.82–4.53) ***
Agree	11.7	21.6	32.8	2.54 (1.64–3.94) ***	4.97 (3.15–7.84) ***
Some children need to be hit so that they will learn a lesson					
Disagree (ref)	93.4	86.3	83.9	1.00	1.00
Neither	4.7	10.0	10.2	2.35 (1.29–4.28) **	2.47 (1.29–4.74) **
Agree	1.9	3.7	5.9	1.82 (0.72–4.59)	2.91 (1.18–7.18) *

*Abbreviations*: *ARRR*  Adjusted relative risk ratio, *CI* Confidence interval, *ARRR* Adjusted for adolescent sex, household income and parent education, *ref*  reference group

^*^ p < .05; ** *p* < .01; *** *p* < .001

Data presented in Table 5 indicates that parental agreement that “spanking is a normal part of parenting” was associated with 16.35 (95% CI = 10.55 to 25.33) times increased odds of parent-reported spanking and 3.71 (95% CI = 2.52 to 5.46) times increased odds of adolescent-reported spanking. Parental agreement that “some children need to be spanked” was associated with 11.62 (95% CI = 7.72 to 17.50) times increased odds of parent-reported spanking and 2.96 (95% CI = 2.04 to 4.31) times increased odds of adolescent-reported spanking. Parental agreement that “some children need to be hit” was associated with 2.71 (95% CI = 1.29 to 5.69) times increased odds of parent-reported spanking.

**Table 5:** Associations between parent beliefs about spanking and parent- and adolescent-reported spanking

Parent-reported Spanking Beliefs	Parent-reported: Child ever spanked, age 10 or younger			Adolescent-reported: Ever spanked, age 10 or younger		
	No %	Yes %	AOR (95% CI)	No %	Yes %	AOR (95% CI)
Spanking is a normal part of parenting						
Disagree (ref)	75.9	24.1	1.00	67.4	40.3	1.00
Neither	16.9	39.7	7.71 (5.35–11.09) ***	20.8	32.7	2.76 (1.96–3.89) ***
Agree	7.2	36.2	16.35 (10.55–25.33) ***	11.8	27.0	3.71 (2.52–5.46) ***
Some children need to be spanked so that they will learn a lesson						
Disagree (ref)	76.8	29.8	1.00	68.4	45.3	1.00
Neither	14.5	33.3	6.43 (4.43–9.33) ***	18.2	28.2	2.59 (1.81–3.69) ***
Agree	8.7	36.9	11.62 (7.72–17.50) ***	13.3	26.5	2.96 (2.04–4.31) ***
Some children need to be hit so that they will learn a lesson						
Disagree (ref)	93.7	83.1	1.00	91.9	85.4	1.00
Neither	4.0	11.5	3.53 (2.04–6.10) ***	4.7	10.9	2.66 (1.54–4.58) ***
Agree	2.3	5.4	2.71 (1.29–5.69) **	3.4	3.7	1.11 (0.53–2.32)

*Abbreviations*: *AOR* Odds ratio adjusted for adolescent sex, household income and parent education, *CI* Confidence interval, *ref* reference group

^*^ p < .05; ** *p* < .01; *** *p* < .001

Table 6 shows the association between parent- and adolescent-reported spanking and the sociodemographic variables. Sex of the adolescent was not associated with parent- or adolescent- reported spanking. Some significant results were found for household income and parent education and parent- as well as adolescent-reported spanking. Household income of $50,000 to $99,999 compared to $150,000 or more was associated with increased odds of parent- (AOR = 1.56; 95% CI = 1.05 to 2.32) and adolescent-reported spanking (AOR = 2.02; 95% CI = 1.35 to 3.01). Parent education of some post-secondary compared to a graduate degree was associated with increased odds of parent-reported spanking (AOR = 1.65; 95% CI = 1.02 to 2.66), while an undergraduate degree compared to a graduate degree was associated with decreased odds of adolescent-reported spanking (AOR = 0.66; 95% CI = 0.45 to 0.97). All other comparisons across other income and education categories were non-significant.

**Table 6:** Adolescent sex, household income, and parent education association with parent- and adolescent-reported spanking

	Parent-reported: Child ever spanked, age 10 or younger	Adolescent-reported: Ever spanked, age 10 or younger
	AOR (95% CI)	AOR (95% CI)
**Adolescent Sex**		
Male	1.16 (0.89–1.51)	0.91 (0.69–1.19)
Female (ref)	1.00	1.00
**Household Income**		
$49,999 or less	1.44 (0.92–2.25)	1.34 (0.85–2.10)
$50,000 to $99,999	1.56 (1.05–2.32) *	2.02 (1.35–3.01) **
$100,000 to $149,999	1.24 (0.81–1.91)	1.36 (0.89–2.09)
$150,000 or more (ref)	1.00	1.00
**Parent Education**		
High school completion or less	1.19 (0.75–1.88)	0.98 (0.62–1.54)
Some post-secondary	1.65 (1.02–2.66) *	1.53 (0.93–2.51)
Completed trade school or community college	1.43 (0.97–2.12)	1.05 (0.71–1.55)
Completed a university undergraduate degree	1.32 (0.90–1.93)	0.66 (0.45–0.97) *
Completed a university graduate degree (ref)	1.00	1.00

*Abbreviations*: *AOR* Adjusted odds ratio, *CI* Confidence interval. Adolescent sex, household income, and parent education were all entered simultaneously in the model, *ref* reference group

^*^ p < .05; ** *p* < .01; *** *p* < .001

## Discussion

Novel findings from this work are as follows. First, the strength of adolescents’ and parents’ agreement that spanking is a normal part of parenting were similar and correlated. Second, proportions, crosstabulations, and correlations indicate that parents perceive the words “spank” and “hit” differently. Third, odds ratios indicated that adolescents’ beliefs that spanking is normative were associated with parents’ positive beliefs about “spanking” or “hitting”. Finally, only a few significant associations were found for sociodemographic variables and parent-reported and adolescent-reported spanking.

The prevalence of spanking by parents during childhood remains high whether it be reported by adolescents (46%) or parents (39.6%). Given the numerous detrimental outcomes linked to corporal punishment, its prevalence highlights the need to strengthen efforts to prevent its use in Canada. More research is needed to inform spanking prevention efforts. Foremost, the finding that adolescents’ and parents’ beliefs were positively correlated suggests cross-generational transmission. Parents’ agreement that spanking is normative was associated with 7.09 times increased odds that adolescents would have the same belief. Parents’ beliefs in the normativity and necessity of spanking were strong predictors of whether their children had been spanked, as reported by either parents or their adolescents.

These findings point to the urgency of shifting beliefs about spanking in Canada. Findings from the current study indicate that the belief that spanking is normative is likely transmitted across generations from parent to child, so efforts to change these beliefs may well reduce intergenerational cycles of violence against children. There is evidence to suggest that prohibiting physical punishment of children is related to decreased support for physical punishment, and can serve to challenge views of the convention and necessity of such practices [47, 48] However, law reform alone is not enough. Prevention efforts that begin during adolescence, before many become parents themselves, may be effective in reducing the belief that spanking is a normal part of parenting. We are not aware of any existing prevention initiatives targeting adolescents. However, results from a recent systematic review of intervention programs designed to promote healthy romantic relationships in youth did show evidence across several studies for changes in beliefs about acceptance of dating violence among those in intervention groups compared to controls [49]. These findings should encourage the development and evaluation of adolescent interventions with the aim of shifting beliefs about spanking.

The current study supported previous findings that parents’ positive spanking beliefs are associated with an increased likelihood of parent-reported spanking [30–32]. Beyond this finding, this study found that parents’ beliefs were also associated with adolescent-reported spanking. Using multiple informants and finding consistent trends in the data extends knowledge and is a strength of this work.

Another finding of this study is that parents’ beliefs about the necessity of physical punishment differed depending on the verb presented. More than five times as many parents agreed that some children need to be spanked than agreed that some children need to be hit. Consistent with previous findings, [43] these parents appear to compartmentalize “spanking” into what they consider to be a less harmful, more acceptable, and perhaps even useful form of violence. Objectively, spanking is hitting; the parent is striking the child. The only difference is in the word used to describe the act. This finding suggests that public education messaging to prevent corporal punishment should target the erasure of this false dichotomy. By making it evident that spanking is indeed a form of hitting, parents might perceive their actions differently and their acceptance of physical punishment should decrease.

Finally, previous research has shown inconsistent relationships between sociodemographic variables and use of spanking [29, 50–53]. The present results indicated that the prevalence of parent- and adolescent-reported spanking was equally common across almost all levels of sociodemographic variables. Only a few significant associations were found for sociodemographic variables and parent-reported and adolescent-reported spanking. Childhood experience of spanking was not associated with sex of the adolescent in either the parent- or adolescent-reported spanking models. These findings do not support implementation of educational prevention efforts targeted to specific groups; our findings support development of universal awareness-raising campaigns and public education strategies in Canada.

These findings should be considered along with the limitations of the study. First, data from this study were cross-sectional, which means inferences regarding causation are not possible. Second, the parents and adolescents were reporting on spanking experiences that occurred at before age 11 years. Recall errors are possible with the retrospective recall of experiences. Third, although this study used a large community sample that is similar to the population from which it was drawn, it may not be representative. Finally, most of the parents included in the study were female. It would have been informative to have had greater representation of fathers or male caregivers or to include all parent/caregivers in the case of multi-parent families.

The current findings have several important clinical and public health implications. First, the high prevalence of spanking calls for strong public education messaging in Canada. All levels of government, professional associations, and individual practitioners need to give a clear and consistent message that all physical punishment places children’s development at risk. To date, more than 660 professional organizations in Canada have endorsed the Joint Statement on Physical Punishment of Children and Youth, [54] which calls for public awareness strategies and for universally available parenting education and information about positive discipline in programs for babysitters, child and youth workers, early childhood educators, and teachers. The number of available parenting programs that are aimed specifically at reducing physical punishment is continually growing [55].

Second, to reduce intergenerational transmission of punitive violence, it is important to change underlying beliefs that influence such behaviours. Canada’s law still justifies physical punishment of children between the ages of 2 and 12 years within particular parameters [56]. This law sends the message that physical punishment is ‘normal’, effective, and sometimes necessary contradicting the efforts of public education initiatives and perpetuating the beliefs that have been repeatedly demonstrated to contribute to punitive violence against children. At the highest level, law reform and policy changes can transform societal norms or normative beliefs [47, 48]. In countries where population-level data are available, prohibitions against physical punishment have been followed by dramatic declines in approval and use of multiple forms of corporal punishment (including “spanking”) [57]. Law reform must be supported by public health intervention strategies such as universal awareness-raising campaigns and consistent public messaging that indicates that children should never be hit [6].

Third, the present findings indicate that it is important for parents to understand that spanking and hitting are the same action – these are both acts of punitive violence. This may help to shift the perception held by many people that spanking is a normal part of child-rearing and help parents to make the decision to learn constructive ways of guiding children’s learning. Raising awareness that spanking is a form of hitting may begin to shift societal norms and decrease tolerance of violence against children in the name of discipline.

Fourth, providing resources to adolescents in school and community settings may also be an important prevention strategy to shift beliefs and increase knowledge about positive parenting before many of these individuals become parents. School- or community-based programs for adolescents should be developed and tested for efficacy. Finally, it is important to communicate these findings to clinicians and other professionals working with parents and families to encourage consistent messaging from clinicians and other professionals that parents should never spank/hit children, which is an important part of shifting societal norms and reducing physical punishment.

## Conclusions

In conclusion, parents’ spanking beliefs are related to children’s experiences of spanking and their own attitudes toward it, contributing to the intergenerational transmission of violence. Similarly, efforts to decrease support for spanking have the potential to reduce it. Educational prevention strategies focused on reducing intergenerational transmission of positive beliefs about spanking hold considerable promise. As well, the present findings suggest that helping parents to understand that “spanking” is actually hitting may be an important component of education programs. Given that few of the sociodemographic variables categories were associated with parent-reported and adolescent-reported spanking along with prevalent use of spanking across all sociodemographic variable categories, efforts are needed to focus on evidence-based universal public health prevention strategies.

## Data Availability

Data are not publicly available. The datasets generated and/or analysed during the current study are not publicly available due to The WE Study data not being anonymous. The data must be housed in a secured lab and cannot be made publicly available because of the sensitive nature of the data and privacy and confidentially guidelines. However, the data could be made available for collaboration from the corresponding author on reasonable request.

## Abbreviations

AOR: Adjusted odds ratio
ARR: Adjusted risk ratio
CI: Confidence intervals
SE: Standard error
The WEStudy: The Well-being and Experiences Study
